# Association Between miR-148a and DNA Methylation Profile in Individuals Exposed to Lead (Pb)

**DOI:** 10.3389/fgene.2021.620744

**Published:** 2021-02-17

**Authors:** Marília Ladeira de Araújo, Bruno Costa Gomes, Paula Pícoli Devóz, Nathália de Assis Aguilar Duarte, Diego Luis Ribeiro, Adriana Ladeira de Araújo, Bruno Lemos Batista, Lusânia Maria Greggi Antunes, Fernando Barbosa, António Sebastião Rodrigues, José Rueff, Gustavo Rafael Mazzaron Barcelos

**Affiliations:** ^1^Department of Biosciences, Institute of Health and Society, Federal University of São Paulo, Santos, Brazil; ^2^Center for Toxicogenomics and Human Health, NOVA Medical School (NMS), Universidade Nova de Lisboa, Lisbon, Portugal; ^3^Department of Clinical Analyses, Toxicology and Food Sciences, School of Pharmaceutical Sciences of Ribeirão Preto, University of São Paulo, Ribeirão Preto, Brazil; ^4^Department of Genetics, Ribeirão Preto Medical School, University of São Paulo, Ribeirão Preto, Brazil; ^5^Anhembi Morumbi University, São Paulo, Brazil; ^6^Center for Natural and Human Sciences, Federal University of ABC, Santo André, Brazil

**Keywords:** epigenetics, miRNA, ncRNA, occupational exposure, toxicity, lead

## Abstract

Experimental and epidemiologic studies have shown that lead (Pb) is able to induce epigenetic modifications, such as changes in DNA methylation profiles, in chromatin remodeling, as well as the expression of non-coding RNAs (ncRNAs). However, very little is known about the interactions between microRNAs (miRNAs) expression and DNA methylation status in individuals exposed to the metal. The aim of the present study was to investigate the impact of hsa-miR-148a expression on DNA methylation status, in 85 workers exposed to Pb. Blood and plasma lead levels (BLL and PLL, respectively) were determined by ICP-MS; expression of the miRNA-148a was quantified by RT-qPCR (TaqMan assay) and assessment of the global DNA methylation profile (by measurement of 5-methylcytosine; % 5-mC) was performed by ELISA. An inverse association was seen between miR-148a and % 5-mC DNA, as a function of BLL and PLL (β = −3.7; *p* = 0.071 and β = −4.1; *p* = 0.049, respectively) adjusted for age, BMI, smoking, and alcohol consumption. Taken together, our study provides further evidence concerning the interactions between DNA methylation profile and miR-148a, in individuals exposed to Pb.

## Introduction

Previous studies have shown that exposure to toxic metals, such as lead (Pb), induces alterations in gene expression, by modulation of epigenetic status and, consequently, may influence Pb-induced toxicity (Senut et al., 2012; Kim et al., 2014; Nye et al., 2014). In this context, it is noteworthy that an increasing number of studies is being conducted to understand the impact of disturbances on epigenetic status induced by Pb exposure, with special focus on DNA methylation and on the expression of non-coding RNAs (nc-RNAs), mainly micro-RNAs (miRNAs).

Earlier epidemiological studies showed associations between Pb exposure and changes in DNA global methylation profiles (Pilsner et al., 2009; Nye et al., 2015; Sen et al., 2015; Devóz et al., 2017). However, the molecular mechanisms underlying these events are still unclear. Experimental evidences give support to the fact that Pb-induced alterations of the activity of DNA methyl transferases (DNMTs) may be related to these observations (Schneider et al., 2013; Sanchez et al., 2017; Sobolewski et al., 2018). DNMTs play a key role in the maintenance of genes’ promoter region methylation, especially in CpG islands. It is well known that hypo- and hypermethylation of CpG islands in promoter regions are associated with an increase or decrease of mRNA transcription, respectively (for a review, see Ehrlich and Lacey, 2013; Moore et al., 2013).

The identification of molecular biomarkers that may precede the adverse health effects related to the exposure of toxicants significantly increased in the last decade. This approach is particularly interesting and cost-effective, since it provides evidence about underlying toxic effects, being possible to draw safety decisions prior to acute toxicity responses (for a review, see Califf, 2018; Turesky and Lu, 2020). In this context, the detection of miRNAs seems to be a promising tool for biomonitoring individuals who are exposed to chemical compounds, such as toxic metals and organic pollutants (Kotsyfakis and Patelarou, 2019; Sisto et al., 2019). Several studies showed clear associations between the expression of miRNAs and the increase in cancers (Sohel, 2020). On the other hand, a few studies have been performed aiming to assess the impact of toxic metals, such as Pb, arsenic (As), cadmium (Cd) and mercury (Hg) on miRNAs expression (Wallace et al., 2020); moreover, most of them were carried out using *in vitro* or *in vivo* laboratory models (Xu et al., 2015; Bihaqi, 2019; Wallace et al., 2020) and fewer studies have assessed the impact of Pb on miRNA expression profile in individuals exposed to the metal.

For example, Xu et al. (2017) observed an inverse association between expression of miR-520c-3p, miR-211, and miR-148a and high blood lead levels (BLL), while miR-572 levels increased in individuals with high BLL, in workers from China. In another study, Kong et al. (2012) assessed the association between the exposure to toxic metals, including Pb and miRNAs expression, in adolescents from Hong Kong, and an inverse association was observed between miR-21 and miR-221 and urinary Pb and As levels, when compared to the non-exposed ones.

As described above, there are few studies focusing on the disturbances of miRNA expressions induced by Pb exposure, in humans. Therefore, the aim of the present study was to investigate the impact of Pb exposure on the expression of miR-148a and its association with DNA methylation, in individuals exposed to metal, from automotive battery plants.

## Materials and Methods

### Population and Study Design

A cross-sectional study with 85 male individuals (>18 years old) was carried out from automotive battery plants, in Paraná State, Brazil. An interviewed-administrated questionnaire was applied in order to collect socio-demographic, lifestyle, and health information, such as age, body mass index (BMI), time of exposure (working time), medical history, medication use, smoking, and alcohol intake. Participants who drank alcoholic beverages at least five times per week were considered alcohol users and those who had smoked at least five cigarettes per day for the previous 5 years were classified as smokers (Barcelos et al., 2013, 2015a, 2015b; de Oliveira et al., 2014; Gomes et al., 2018).

The present study was approved by the Ethics Committee of University Federal of São Paulo, Santos, Brazil (approval number: 0292/2018), and the corresponding methods were carried out in accordance with the approved guidelines. All participating workers were advised about the content of the investigation and signed the written informed consents before starting the study.

### Quantification of Pb in Blood and in Plasma

Samples were taken on site, in the infirmary station of each plant. Blood samples were collected by a qualified nurse using evacuated tubes: (I) for Pb determination and DNA isolation: Vacutainer Trace-Elements and Vacutainer PST (BD, Franklin Lakes, NJ, United States) and (II) for miRNA isolation: PAXgene Blood RNA Tubes (PreAnalytiX, Hombrechtikon, Switzerland); plasma samples were obtained by centrifugation (10 min at 1,200 *g*). Transportation of the samples was carried out using Styrofoam boxes with dry ice until they arrived at the laboratory; samples were kept at −80°C till further handling and analyses.

Total BLL and PLL levels were determined by inductively coupled plasma mass spectrometry (ICP-MS; ELAN DRC II, Perkin Elmer, Norwalk, CT, United States) as previously described by Batista et al., 2009a, b). Results are expressed as μg dl^–1^.

### hsa-miR-148a-5p Expression Assessment

Total RNA was extracted from whole blood using MagMax for Stabilized Blood Tubes RNA Isolation Kit (Applied Biosystems, Foster City, CA, United States) according to the manufacturer’s instructions. The quality of the RNA was verified by measuring the 260/280 and 260/230 nm ratio (Nanodrop 2000, Invitrogen, California, CA, United States). Samples were quantified using Qubit RNA BR Assay Kit (Invitrogen, California, CA, United States) on a fluorimeter (Quibit 3.0, Invitrogen, California, CA, United States), according to the manufacturer’s recommendations.

TaqMan Advanced miRNA Assay (Applied Biosystems, Foster City, CA, United States) was used for cDNA synthesis. Monitoring of miR-148a expression was assessed using TaqMan Advanced miRNA Assay (assay #478718_mir; Applied Biosystems, Foster City, CA, United States), according to the manufacturer’s instructions; moreover, quantification of expression of has-miRNA-miR-16-5p was used as an endogenous control (assay #477860_mir; Applied Biosystems, Foster City, CA, United States). miRNAs were quantified using the relative quantification method [2^–(Δ^ ^Ctx – Δ^ ^Ctr)^ = 2^–Δ^ ^Ct^]. All RT-qPCR reactions were performed in a QuantStudio 3 Real Time PCR System Thermal Cycler (Applied Biosystems, Foster City, CA, United States).

### Global Methylation Assays

Genomic DNA (gDNA) was extracted from peripheral blood using the ReliaPrep Blood gDNA Miniprep System (Promega, Wisconsin, WI, EUA) according to the manufacturer’s instructions. The quality of the DNA was verified by measuring the 260/280 and 260/230 nm ratio (Nanodrop 2000, Invitrogen, California, CA, United States). Subsequently, gDNA was quantified by use of the Qubit dsDNA BR Assay Kit (Invitrogen, California, CA, United States) in a fluorimeter (Qubit 3.0, Invitrogen, California, CA, United States).

Quantification of the global DNA methylation status was performed using the 5-mC DNA ELISA Kit (Zymo Research, Irvine, CA, United States), according to the manufacturer’s recommendations; absorbance was read at 405 nm (Biotek Elx800—Winooski, VT, United States). Results are expressed as % DNA global methylation (% 5-mC DNA).

### Data Interpretation

Age (years), body mass index (BMI), BLL, PLL, miR-148a expression, and % 5-mC DNA were analyzed as continuous variables; alcohol consumption (yes or no) and smoking (yes or no) were assessed as categorical ones. Due to their skewed distribution, BLL and BMI were sqrt transformed, while PLL data were log10-transformed.

Descriptive statistics were run for reporting the general characteristics of the participants. Non-parametric correlations (Spearman’s rho) were performed in order to examine the associations between age, BMI, alcohol consumption, smoking, exposure period, BLL, PLL, % 5-mC DNA, and miR-148. Then, multivariate linear regression models were performed to assess the associations of Pb biomarkers, miR-148a and % 5-mC DNA, adjusted for age, BMI, alcohol consumption, and smoking.

Analyses were run using SPSS 23 Statistics software (IBM; Armonk, NY, United States), and a *p* < 0.050 was set as significant.

## Results

The general population characteristics, Pb concentrations, and % 5-mC DNA for all participants enrolled in the present study are summarized in Table 1. The age ranged from 19 to 69 years (mean 37 ± 11 years), while the mean exposure period was 3.2 ± 3.4 years (from 2 months to 20 years). Alcohol was consumed by 31% of the participants, and 12% of the individuals were declared as smokers. Mean BLL was 19 ± 11 μg dl^–1^ (ranging from 2.6 to 48 μg dl^–1^), and mean PLL was 0.54 ± 0.65 μg dl^–1^, reaching values up to 4.0 μg dl^–1^.

**TABLE 1:** General characteristics of study population.

	*N*	Mean ± *SD*	Median	Range
Age (years)	85	37 ± 11	35	19–69
Exposure period (years)	85	3.2 ± 3.4	1.7	0.2–20
BMI^a^ [kg (m^2^)^–1^]	85	27 ± 3.9	26	18–39
Smoking (yes)	85 (10)	–	–	–
Alcohol (yes)	85 (26)	–	–	–
Pb in blood (μg dl^–1^)	85	19 ± 11	17	2.6–48
Pb in plasma (μg dl^–1^)	85	0.54 ± 0.65	0.35	0.018–4.0
% 5-mC DNA^b^	85	3.0 ± 1.0	3.0	5.3–1.1

*^*a*^body mass index; ^*b*^% of methylcytosine in genomic DNA.*

Table 2 presents the Spearman’s correlations between age, BMI, alcohol consumption, smoking, biomarkers of Pb exposure, % 5-mC, and miR-148a expression. As expected, BLL and PLL were strongly correlated to each other (*p* < 0.0010); also, a significant correlation was seen between age and BMI (*p* = 0.010). On the other hand, none of the assessed variables were significantly correlated with any of the biomarkers related to metal exposure (*p* > 0.050).

**TABLE 2:** Spearman’s correlations between age, body mass index (BMI), alcohol consumption, smoking, working time, blood and plasma lead levels (BLL and PLL, respectively), % DNA global methylation (% 5-mC), and expression of miR-148a.

	Age	BMI	Alcohol	Smoking	BLL	PLL	% 5-mC	miR-148a
Age^a^	–							
BMI^b^	0.28*	–						
Alcohol^c^	0.12	0.12	–					
Smoking^d^	0.018	0.085	0.069	–				
BLL^e^	−0.087	−0.21	0.016	−0.021	–			
PLL^e^	−0.039	−0.21	−0.061	0.080	0.86**	–		
% 5-mC^f^	−0.10	0.10	0.022	0.039	−0.050	−0.086	–	
miR-148a^g^	−0.062	−0.031	0.069	0.053	0.070	0.053	−0.099	–

^*a*^years; ^*b*^kg (m^2^)^–1^; ^C^no alcohol consumption was taken as reference; ^ d^non-smokers were taken as reference; ^*e*^μg dl^–1^; ^*f*^% of methylcytosine in genomic DNA; ^*g*^relative expression of miR-148a. *p < 0.050; **p < 0.010.

Tables 3, 4 summarize the impact of miR-148a expression on % 5-mC DNA through data obtained by multivariate linear regression analyses, adjusted by age, BMI, alcohol consumption, smoking, and BLL or PLL. It can be seen that the content of 5-mC DNA tended to decrease as the miR-148a expression increases, as a function of BLL (β = −3.7; *p* = 0.071); however, this observation did not reach statistical significance. Further, it can be observed that miR-148a expression is able to decrease the % 5-mC DNA, as a function of PLL (β = −4.1; *p* = 0.049).

**TABLE 3:** Impact of miR-148a expression on % DNA global methylation (% 5-mC DNA) of Pb-exposed workers from automotive battery factories. Adjusted for age, BMI, alcohol consumption, smoking, and blood lead levels (BLL).

Variables	% 5-mC DNA		
	β^c^	*p*	95% CI
Age	−0.014	0.23	−0.30; 0.0090
BMI^a^	0.020	0.55	−0.48; 0.89
Alcohol^b^			
No	−0.12	0.65	−0.66; 0.42
Yes	–	–	
Smoking^b^			
No	−0.13	0.72	−0.88; 0.61
Yes	–	–	
BLL^a^	−0.023	0.44	−0.83; 0.037
miR-148a	−3.7	0.071	−7.8; 0.32

*^*a*^sqrt-transformed; ^*b*^yes was taken as reference; ^C^unstandardized beta (β) coefficients for covariates in the model. Math model: % 5-mC DNA = α + β1 × age + β2 × BMI + β3 × alcohol + β4 × smoking + β5 × BLL + β6 × miR-148a.*

**TABLE 4:** Impact of miR-148a expression on % DNA global methylation (% 5-mC DNA) of Pb-exposed workers from automotive battery factories. Adjusted for age, BMI, alcohol consumption, smoking, and plasma lead levels(PLL).

Variables	% 5-mC DNA		
	β^d^	*p*	95% CI
Age	−0.015	0.21	−0.038; 0.0080
BMI^a^	0.016	0.65	−0.52; 0.84
Alcohol^b^			
No	−0.10	0.70	−0.64; 0.43
Yes	–	–	–
Smoking^b^			
No	−0.18	0.62	−0.93; 0.53
Yes	–	–	–
PLL^c^	−0.38	0.15	−0.89; 0.14
miR-148a	−4.1	0.049	−8.1; −0.010

*^*a*^sqrt-transformed; ^*b*^yes was taken as reference; ^*C*^log10-transformed; ^*d*^unstandardized beta (β) coefficients for covariates in the model. Math model: % 5-mC DNA = α + β1 × age + β2 × BMI + β3 × alcohol + β4 × smoking + β5 × PLL + β6 × miR-148a.*

Finally, no effects of other variables (age, BMI, alcohol intake, and smoking) were seen on % 5-mC DNA (Tables 3, 4), and no significant interaction terms (IT) between the biomarkers of Pb exposure and miR-148a were able to induce disturbances of DNA methylation profile (IT for BLL^∗^miR-148a: β = 0.43; *p* = 0.45; IT for PLL^∗^miR-148a: β = 2.3; *p* = 0.54; not in Tables).

## Discussion

It is well stablished that Pb exposure is able to induce several adverse health effects, such as cardiovascular disorders (Lustberg and Silbergeld, 2002), kidney injuries (Weaver et al., 2005), and cognitive dysfunctions (Shih et al., 2007); previous data showed that BLL as low as 10 μg dl^–1^ can cause hypertension, tremors, and renal dysfunction (National Toxicology Program, 2012), and BLL up to 20 μg dl^–1^ induces several neurological injuries, in adults (Murata et al., 2009). Moreover, recent studies suggest that exposure to the metal is associated with changes in epigenetic status and, consequently, may modulate the toxic effects related to Pb exposure (Senut et al., 2012; Kim et al., 2014; Nye et al., 2014).

We previously showed that Pb exposure is able to decrease the DNA global methylation profile of leukocytes from peripheral blood, in a subgroup of this study population (Devóz et al., 2017). Moreover, other studies also showed the impact of Pb-induced disturbances on the DNA methylation profile. For example, Zhang et al. (2019) observed alterations in DNA methylation profile of 356 significant CpG sites of blood cells, and these changes were related to the levels of exposure to the metal, in workers from car battery facilities, in China; Li et al. (2013) demonstrated that individuals exposed to Pb levels similar to those found in our study (21 μg dl^–1^) had lower % of DNA methylation assessed by LINE-1 than the non-exposed ones (3.7 μg dl^–1^). Besides, associations between Pb exposure and DNA hypomethylation were also seen in animal laboratory models (Dou et al., 2019; Nakayama et al., 2019).

Studies performed with laboratory models showed that Pb is able to inhibit the DNA methyl transferase (Dnmts) activities. For example, Sanchez et al. (2017) observed that zebra fish exposed to the metal throughout embryogenesis (500 ppb, i.e., 500 μg l^–1^) had lower Dnmt1 activity than non-exposed animals, while Nakayama et al. (2019) showed that Wistar rats exposed to Pb- and Cd-contaminated soil (3,750 and 6.0 mg kg^–1^ bw, respectively) had higher Dnmt3a and Dnmt3b mRNA expression in testis than the control subjects (Nakayama et al., 2019).

miRNAs are being widely used as epigenetic biomarkers, since they canregulate various cellular and molecular pathways (Gholamin et al., 2018; Rabieian et al., 2018). Currently, these biomarkers are not limited to diagnosis or to therapeutic monitoring response; miRNAs are also used as predictive tools for underlying adverse health effects induced by exposure to toxicants, predicting by molecular signaling the onset of pathologies and prior to manifestation of symptoms and complications. For example, miRNAs are widely used as a prognostic biomarker for myocardial infarction (Wang et al., 2016), and for type 1 and 2 diabetes (Nielsen et al., 2012; Villard et al., 2015; Marchand et al., 2016). The monitoring of miRNAs as predictive tools is particularly advantageous, due their non-invasive collection (mostly plasma and saliva) and good correlation to disorders of several inner organs and systems (Condrat et al., 2020).

Alterations of miRNA expression profile can be a sensitive indicator related to acute and/or chronic exposures to several inorganic and organic toxicants. Chronic Pb poisoning is a complex disease, due to interactions between genetic backgrounds and environmental variables, and also because of the long latency between the beginning of exposure to the metal and the onset of adverse health effects (Mani et al., 2019). In this context, biomonitoring alterations of miRNA expression may be a useful tool for predicting cellular and systemic response against Pb-induced toxicity (for a comprehensive review, see Wallace et al., 2020).

We observed an inverse association between miR-148a expression and % 5-mC DNA. Data from *in silico* and from *in vitro* studies showed that miR-148a has the *DNMT1* gene as target (Long et al., 2014; Zhan et al., 2015; Sengupta et al., 2018). *DNMT1* plays a key role on the maintenance of genes’ promoter region methylation, especially in CpG islands. It is well-known that hypo- and hypermethylation of CpG sites in promoter regions are associated to an increase and decrease in mRNA translation, respectively (for a review, see Ehrlich and Lacey, 2013; Moore et al., 2013). One hypothesis of our findings may be explained to the premature degradation of *DNMT1* mRNAs induced by overexpression of miR-148a, which would impact the global DNA methylation status.

Previous studies give further support to this explanation; Lujambio et al. (2008) showed that miR-148a silencing was associated with DNA hypermethylation in different metastatic cell lines, while Sengupta et al. (2018) observed that miR-148a suppressed *DNMT1* expression, in prostate cancer cells. Moreover, Wang et al. (2019) found similar results of those previously reported by Lujambio et al. (2008) and Sengupta et al. (2018), in an acute myeloid leukemia cell line, i.e., the increase in miR-148a decreases the expression of *DNMT1* (both mRNA and protein), which may impact the DNA methylation profile, suggesting that lower DNMT1 activity is related to a decrease in the methylation levels of promoter region’s CpG islands of miR-148a and, consequently, increases miR-148a expression, creating a negative feedback system. It is important to note that these studies were carried out in cancer cell lines, and the mechanisms may differ in normal cells, as well as in complex organisms, such as mammals and humans, for example.

To the best of our knowledge, we have found a few studies related to Pb exposure and miRNA expression in humans, suggesting that the metal is able to induce disturbances on miRNA expression profiles. For example, in a study conducted with workers from battery car plants in China, it was seen that individuals who had higher BLL (higher exposure: BLL mean 51 ± 6.4 μg dl^–1^) had lower expression of plasmatic miRNAs miR-520c-3p, miR-211, and miR-148a, when compared to those that were categorized as lower exposure persons (BLL 8.9 ± 1.5 μg dl^–1^), while opposite effect was seen concerning miR-572 (Xu et al., 2017). In contrast, we did not find a significant association between BLL and PLL, and miR-148a expression. These contradictory results may be partly explained by differences of studied populations, such as variation on genetic background, dietary habits, and lifestyle; moreover, it is important to highlight that the level of Pb exposure in the study carried out by Xu et al. (2017) is much higher than those found in our study (BLL mean 51 ± 6.4 vs. 19 ± 11 μg dl^–1^, respectively) suggesting that high Pb levels may trigger metal–epigenetic interactions.

Taken together, our study provides further evidence concerning decrease in DNA global methylation induced by miR-148a in workers exposed to Pb. The consequences may result in impairment in the regulation of gene expression and, consequently, modulate the adverse health effects induced by exposure to the metal.

## Data Availability Statement

The raw data supporting the conclusions of this article will be made available by the authors, without undue reservation.

## Ethics Statement

The studies involving human participants were reviewed and approved by the Ethics Committee of Federal University of São Paulo, São Paulo, Brazil. The patients/participants provided their written informed consent to participate in this study.

## Author Contributions

AR, GB, JR, and MA conceptualized the study. BG and MA developed the methodology and performed the miRNA assays. BB and FB performed the ICP-MS analysis. DR, LA, and PD performed the global methylation assays. GB, MA, and ND conducted the investigation. AR, GB, and JR wrote and prepared the original draft. AR, GB, JR, MA, and ND wrote reviewed, and edited the manuscript. All authors have read and agreed to the published version of the manuscript.

## Conflict of Interest

The authors declare that the research was conducted in the absence of any commercial or financial relationships that could be construed as a potential conflict of interest.
